# Supplementary material for: ‘It is a disease which comes and kills directly’: What refugees know about COVID-19 and key influences of compliance with preventive measures
Source: PLoS One. 2021 Dec 21;16(12):e0261359. doi: 10.1371/journal.pone.0261359 (PMC8691645; doi:10.1371/journal.pone.0261359)
Supplement: Attachment — Submitted filename: PLOS ONE review Oct 2021.pdf [file pone.0261359.s002.pdf]

'It is a Disease which comes and kills directly'...key influences of compliance with preventative measures.

Comments by Dr.Siyana Mahroof-Shaffi

47

It is questionable whether high mobility is an issue during various lockdowns. Our experience was that the refugee camps were the first to become 'locked' with limited movement in and out of the camps. Elaborate on issues such as poor hygiene in facilities/ access to WASH facilities and probable overcrowding.

Ref: (https://via.hypothes.is/https://www.medrxiv.org/content/10.1101/2020.07.07.20140996v2#annotations:Q3NC_sCNEeqwwYO0MegHbw).ef Tucker et al:

111 (Study population)

Very Low numbers already acknowledged. It would be good to understand how this compares to the size of the population being studied. How many in total are in the area? Also men vs women?

244

Typo= 'visited"

370-401

This section could be perceived as being quite biased. It is based on the commentary of three community workers. It would be interesting to know which faith background the workers come from?

I agree that it contrasts with the interviews with the patients themselves were far less loaded. I would suggest taking this section out as it seems unnecessarily inflammatory. In general there has been a lot of blame COVID (eg US blaming China...then China laughing as US cases soared). The take home message instead could be that despite all our technological and scientific success- there has been no rational explanation for the onset of this pandemic. More than one faith followers have expressed the phenomenon of this being a sign of the 'end of the world' and so it seems a bit harsh to penalise just the Muslims.

In UK for example, various communities had continued to gather (eg Jewish wedding/ secret parties amongst youngsters)...ultimately the focus has to be on ignorance or defiance which can affect any group of people and not limited to certain faiths only.

517-518

I rest my case…! This study cannot conclude anything based on the small and select group of interviewees.
